# Effect of sugarcane cystatin CaneCPI-5 on osteogenic differentiation of human dental pulp cells

**DOI:** 10.1590/0103-644020256174

**Published:** 2025-10-24

**Authors:** Ana Flávia Balestrero Cassiano, Eduardo Pereira de Souza, Hernán Coaguila-Llerena, Flávio Henrique-Silva, Gisele Faria

**Affiliations:** 1 São Paulo State University (UNESP), School of Dentistry, Araraquara, Department of Restorative Dentistry, São Paulo, Brazil.; 2 Federal University of Sao Carlos (UFSCAR), Department of Genetics and Evolution, São Paulo, Brazil.; 3 Cayetano Heredia Peruvian University (UPCH), School of Stomatology, Department of Endodontics, Lima, Peru.

**Keywords:** CaneCPI-5, Cell differentiation, Dental pulp cells, Phytocystatins

## Abstract

For pulp and periapical repair, or endodontic regeneration to occur, it is necessary for mesenchymal stem cells from the apical papilla and/or dental pulp to proliferate, migrate to the site of injury, and differentiate into cells that produce mineralized tissue. Therefore, materials used in endodontic therapy should stimulate those events. Phytocystatins are plant-derived cystatins capable of inhibiting cathepsins. Some of them are produced recombinantly, such as CaneCPI-5 (derived from sugar cane). Considering their pro-osteogenic potential, this study aimed to assess the cytocompatibility and effect of CaneCPI-5 on the proliferation, migration, and osteogenic differentiation of human dental pulp stem cells (hDPSCs). The hDPSCs exposed to CaneCPI-5 and unexposed (control) were evaluated as follows: cell viability, by alamarBlue assay; proliferation, by bromodeoxyuridine (BrdU) incorporation assay; migration, by transwell assay; deposition of mineralized nodules, by alizarin red staining; and activity of tissue-nonspecific alkaline phosphatase (TNAP). Data were evaluated by one- or two-way ANOVA, followed by Tukey or Kruskal-Wallis post-test and Dunn or Mann Whitney post-test. CaneCPI-5 was cytocompatible and induced higher migration, proliferation, formation of mineralized nodules, and TNAP activity in hDPSCs compared to control. In conclusion, CaneCPI-5 represents a molecule with promising application in endodontic therapy to stimulate events necessary for pulp and periapical repair/regeneration.

## Resumo

Para que ocorra a reparação pulpar e periapical ou a regeneração endodôntica, é necessário que células-tronco mesenquimais da papila apical e/ou da polpa dentária proliferem, migrem para o local da lesão e se diferenciem em células capazes de produzir tecido mineralizado. Por isso, é desejável que os materiais utilizados na terapia endodôntica estimulem esses eventos. As fitocistatinas são cistatinas de origem vegetal capazes de inibir catepsinas. Algumas delas são produzidas de forma recombinante, como a CaneCPI-5 (derivada da cana-de-açúcar). Considerando seu potencial pró-osteogênico, este estudo teve como objetivo avaliar a citocompatibilidade e o efeito da CaneCPI-5 na proliferação, migração e diferenciação osteogênica de células-tronco da polpa dentária humana (hDPSCs). As hDPSCs expostas à CaneCPI-5 e não expostas (controle) foram avaliados da seguinte forma: viabilidade celular, pelo ensaio alamarBlue; proliferação, pelo ensaio de incorporação de bromodeoxiuridina (BrdU); migração, pelo ensaio Transwell; deposição de nódulos mineralizados, pela coloração com vermelho de alizarina; e atividade da fosfatase alcalina tecidual inespecífica (TNAP). Os dados foram analisados pelo teste ANOVA de uma ou duas vias, seguido do pós-teste de Tukey ou Kruskal-Wallis e pós-teste de Dunn ou Mann Whitney. CaneCPI-5 foi citocompatível, induziu maior migração, proliferação, formação de nódulos mineralizados e atividade de TNAP em hDPSCs em comparação ao controle. Em conclusão, CaneCPI-5 representa uma molécula com aplicação promissora na terapia endodôntica para estimular eventos necessários no reparo/regeneração pulpar e periapical.

**Figure:**
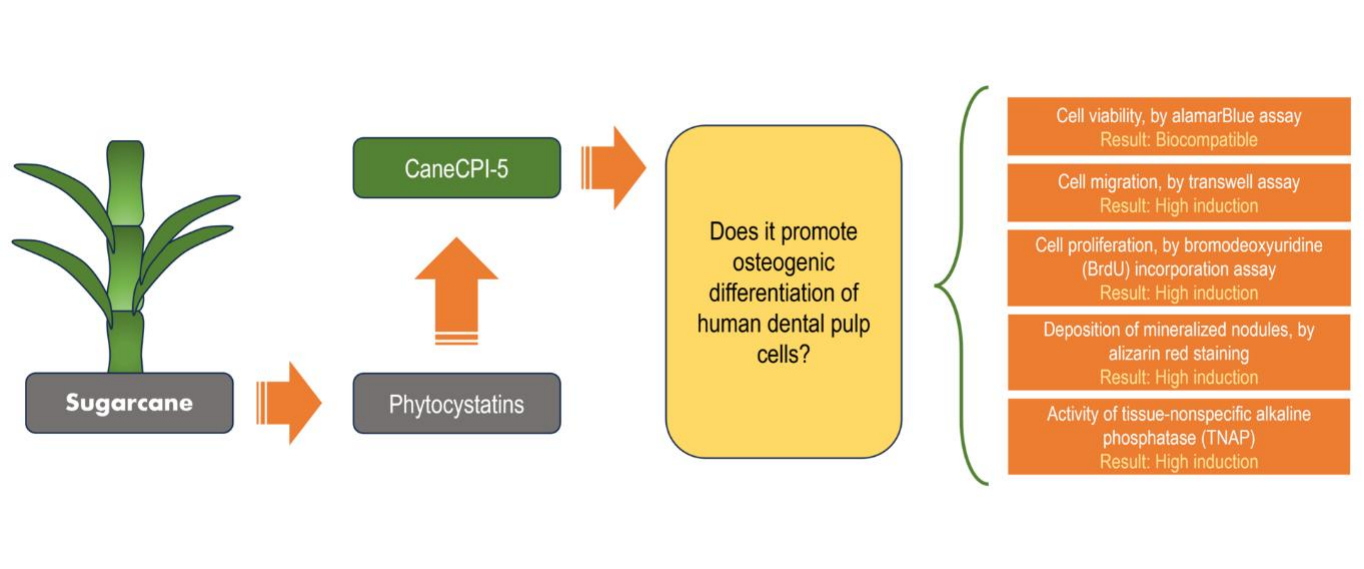

## Introduction

Plant-derived cystatins, mainly angiosperms such as orange and sugar cane are called phytocystatins ^1^. They are involved in the regulation of endogenous proteases in plants ^2^, and some of them have been produced recombinantly. Currently, there are six recombinant cystatins derived from sugar cane, CaneCPI-1 to CaneCPI-6, with a variety of effects and biotechnological applications ^3^.

CaneCPI-5, a potent inhibitor of human cathepsins K, B, and L ^4^, presented favorable results for tissue repair through the reduction of implant-induced inflammation of sponge in mice subcutaneous tissue, promoting angiogenesis and fibrinogenesis ^5^. In Dentistry, CaneCPI-5 reduced and prevented erosion of dental enamel under acid challenging environment, both in vitro and in vivo, by interacting with acquired pellicle ^6^ ^,^ ^7^. More recent studies showed that CaneCPI-5 in association with a chitosan gel prevented erosive dentin or enamel wear in situ ^8^ ^,^ ^9^. Furthermore, CaneCPI-5 reduced biofilm activity and mineral loss from tooth enamel ^10^. On the other hand, it did not change the characteristics and viability of the biofilm on dentin ^11^. Additionally, CaneCPI-5 showed a pro-osteogenic effect on MC3T3-E1 pre-osteoblastic cells ^12^.

In addition to CaneCPI-5, our research group has been researching a recombinant cystatin derived from *Citrus sinensis* or sweet orange, called C*sin*CPI-2, which can inhibit the activity and gene expression of human cathepsins B and K. It has shown anti-inflammatory potential in vitro and in vivo ^1^, and capability to induce a mineralizing phenotype ^13^. In another study, C*sin*CPI-2 used systemically, prevented bone loss induced by periodontal disease in mice by reducing inflammation and osteoclastogenesis ^14^.

For pulp and periapical repair, or endodontic regeneration to occur, mesenchymal stem cells from the apical papilla and/or dental pulp must proliferate, migrate to the site of injury, and differentiate into cells that produce mineralized tissue ^15^. Therefore, materials used in endodontic therapy should stimulate those events ^16^. Given the potential of specific recombinant phytocystatins, such as C*sin*CPI-2 and CaneCPI-5, to induce an osteogenic phenotype ^1^ ^,^ ^12^ ^,^ ^13^, this study aimed to assess the cytocompatibility and the effect of CaneCPI-5 on the proliferation, migration, and osteogenic differentiation of human dental pulp mesenchymal stem cells (hDPSCs). The null hypothesis is that CaneCPI-5 would not promote effects on any of the parameters evaluated.

## Materials and methods

### Expression and purification of the recombinant CaneCPI-5 protein

The target protein of this study was produced recombinantly as previously described ^4^.

### Cell culture

Cells from the batch previously collected and characterized by Cassiano et al. ^15^ were used. Briefly, following faculty ethics committee approval (CAAE: 36595920.7.0000.5416), pulp tissue from healthy third molars of three young donors was collected for hDPSCs primary culture using the enzymatic dissociation method ^15^. A pool of cells from the three donors was cultured in α-MEM (Sigma-Aldrich, St. Louis, MO, USA) with penicillin (100 IU/mL), streptomycin (100 μg/mL) (Gibco/Life Technologies, Grand Island, NY, USA), and 10% fetal bovine serum - FBS (Gibco/Life Technologies). Assays were conducted using cells from the 3^rd^ to 6^th^ passage.

### Immunophenotypic characterization of dental pulp cell culture

Human dental pulp cell culture underwent flow cytometry analysis using mesenchymal (CD146, CD90, CD105, CD73) and hematopoietic/endothelial (CD45, CD34) stem cell markers (BD Biosciences, Pharmingen, San Jose, CA, USA). Cells (3^rd^ passage) were incubated with mouse anti-human antibodies conjugated to the fluorochromes phycoerythrin (PE) or fluorescein isothiocyanate (FITC) and analyzed using a fluorescence-activated cell separator - FACS (BD Biosciences FACS Verse 4C, Pharmingen) as shown in the study by Cassiano et al. ^15^. Afterwards, all tests were performed in triplicate at three different times.

### Cell viability by alamarBlue assay

The cytotoxicity assay was based on the ISO 10993-5:2009 standard. Some guidelines from this standard were also followed in other assays of the present study ^17^. hDPSCs were cultured in 96-well plates (1x10^4^ cells/well) and exposed to CaneCPI-5 at concentrations of 0.0125 μg/uL, 0.025 μg/uL, 0.05 μg/uL, 0.1 μg/uL, 0.2 μg/uL, or α-MEM culture medium (negative control) for 24 and 48 hours (independent assays). Subsequently, cells were incubated with alamarBlue reagent (10:1, Invitrogen, Life Technologies) for 3h at 37°C and 5% CO_2_. Then, 100 µL of the supernatant was transferred to another plate for reading in a fluorescence reader at 560 nm excitation and 590 nm emission wavelengths (Synergy H1, BioTek, Winooski, VT, USA). The percentage of cell viability was calculated considering the negative control as 100% cell viability.

### Cell proliferation assay

The immunosorbent assay for bromodeoxyuridine (BrdU) incorporation utilized the ELISA kit (Roche, Heidelberg, Germany). Following a 24h culture (1.5x10^4^ cells/well in a 96-well plate), cells were treated with CaneCPI-5 (0.05 μg/μL) or α-MEM culture medium for 24h (plate 1) and 48h (plate 2). Subsequently, cells were submitted to the manufacturer's recommendations. Results were presented as a percentage of the CaneCPI-5 group relative to the control group.

### Transwell cell migration assay

hDPSCs (3x10^4^ cells/well) were plated in the upper compartment of a transwell insert (8μm pore, Corning) with α-MEM supplemented with 1% FBS. In the lower compartment, α-MEM supplemented with 1% SBF in a mixture with CaneCPI-5 (0.05 μg/μL) or untreated culture medium (control) was placed; four transwells per group were used. After 24h incubation, cells in the upper compartment were removed. Those that migrated to the membrane were fixed with 4% paraformaldehyde for 10 min, stained with DAPI (1 μg/mL) (Life Technologies), and imaged under a fluorescence microscope (EVOS Fl microscope, AMC, Bothell, WA, USA). ImageJ software (National Institute of Health-NIH, Bethesda, MD, USA) was then used to quantify the migrated cells.

### Alizarin red staining assay

Cells (5x10³ cells/well) were plated in a 24-well plate and exposed to CaneCPI-5 (0.05 μg/μL) diluted in osteogenic medium [α-MEM with ascorbic acid (50 μg/mL) and ß-glycerophosphate (10mM) (Sigma Aldrich)] or osteogenic medium (control). After 21 days, cells were fixed with 70% ethanol at 4°C and stained with 40mM alizarin red solution (pH 4.2; Sigma-Aldrich). The mineralized matrix was visualized on a stereomicroscope (SZ2-ILST, Olympus, Tokyo, Japan) coupled to a camera (E-330, Olympus). Subsequently, a 10% cetylpyridinium chloride solution (Sigma Aldrich) solubilized the matrix. Absorbance was read in a spectrophotometer at 562nm (Asys-UVM 340, Biochrom - Mikro Win 2000). Results were expressed as the percentage of the CaneCPI-5 group relative to the osteogenic control, considered as 100%.

### Activity of tissue-nonspecific alkaline phosphatase (TNAP)

Cells (2x10^4^ cells/well) were plated in 24-well plates and exposed to CaneCPI-5 (0.05 μg/μL) diluted in osteogenic medium, osteogenic medium (positive control), or α-MEM medium (negative control) for 7 days. Cells were washed with stock buffer (50mM Tris and 2mM MgCl_2_, pH 7.5), followed by the addition of osmotic shock buffer (50mM Tris-HCl, 10mM MgSO_4_, and 0.8M NaCl, pH 7.5) for cell collection and lysis. The cell suspension underwent homogenization and ultracentrifugation at 100,000xG for 1 hour at 4ºC. The pellet was homogenized in the stock buffer. Aliquots of this suspension, containing membrane-bound alkaline phosphatase, were added to the reaction medium (aqueous solution with 70mM 2-amino-2-methyl-propan-1-ol (AMPOL) pH 10, 10mM p-nitrophenyl-phosphate (pNPP), and 2mM MgCl_2_). Reactions were halted at the same time intervals. TNAP catalytic activity, measured as p-nitrophenolate (pNP-) formation, was monitored at 410nm using a spectrophotometer (Spectronic Genesys 2). The concentration of pNP- was calculated using Lambert Beer's law ^18^. Data were normalized by total protein concentration via the Hartree method ^19^ and expressed as a percentage, with the osteogenic medium considered as 100%.

### Statistical analysis

The data, resulting of 4 to 6 samples obtained from 3 independent experiments, were analyzed using the statistical software GraphPadPrism 9 (GraphPad Software Inc., San Diego, CA), at 5% significance level. Data were evaluated by one- or two-way ANOVA, followed by Tukey or Kruskal-Wallis and Dunn post-test or Mann Whitney test.

## Results

### Immunophenotypic characterization of hDPSC cultures

hDPSCs expressed high percentages of mesenchymal stem cell markers: CD105 (83.1%), CD90 (95.4%), CD73 (98.5%), CD146 (90.0%); and low percentages of the hematopoietic cell markers: CD45 (0.2%) and CD34 (3.3%), as previously reported in another study from our research group ^15^.

### Cell viability

**Figure 1:**
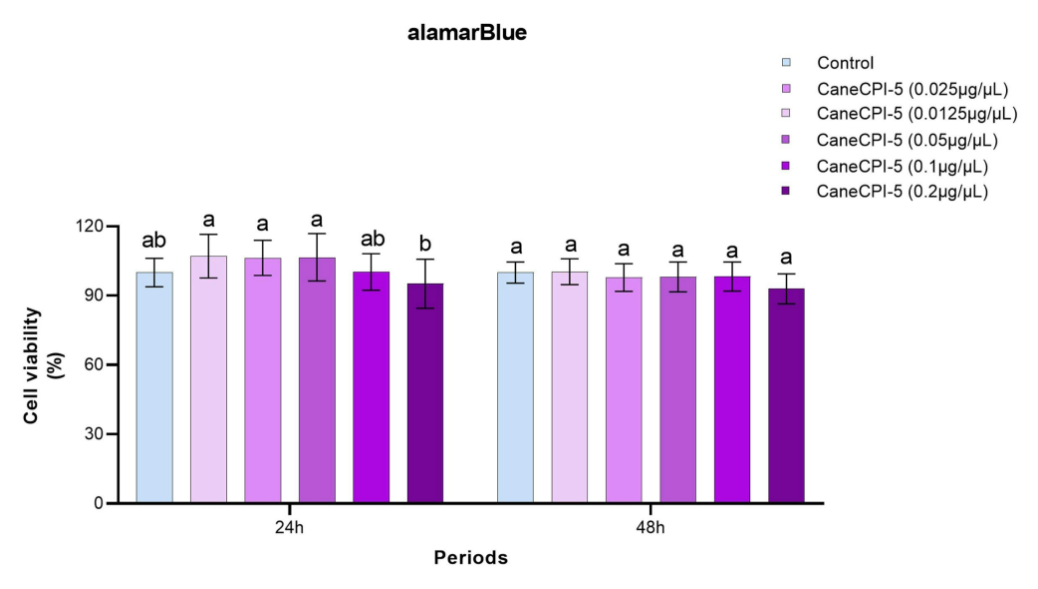
AlamarBlue test. Viability of hDPSCs after exposure to different concentrations of CaneCPI-5 and control group, at 24h and 48h. Different letters in columns indicate significant differences among groups (two-way ANOVA and Tukey post-test).

At 24 and 48h, the alamarBlue assay showed no significant differences between CaneCPI-5 in contact with hDPSCs and the control group (*p* > 0.05). Higher viability of hDPSCs exposed to CaneCPI-5 was observed at 0.0125μg/μL, 0.025μg/μL, and 0.05μg/μL, in comparison to 0.2μg/μL (p<0.05), at 24 hours. There were no significant differences among groups at 48h (p>0.05) (Figure 1).

### Cell proliferation assay

**Figure 2:**
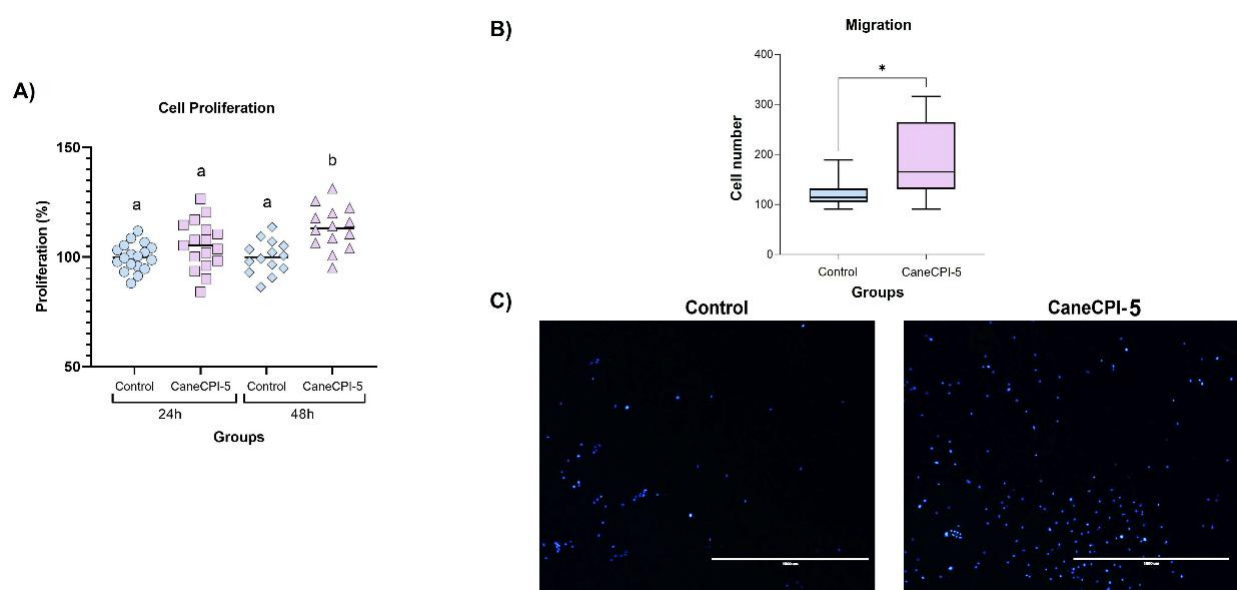
Cell proliferation assay (A) and Cell migration assay (B and C). (A) The proliferation of hDPSCs was assessed by BrdU incorporation assay in control and CaneCPI-5 groups (0.05 μg/μL) for 24h and 48h. Different letters indicate a significant difference between groups (Kruskal-Wallis and Dunn test). (B) Comparison of cell migration by transwell assay. The asterisk (*) indicates significant differences between groups (Mann-Whitney test). (C) Representative images of the nuclei of human dental pulp stem cells stained with DAPI that migrated through the transwell membrane (blue fluorescence of the nuclei), in control and CaneCPI-5 (0.05 μg/μL) groups. Bar = 1000μm.

As seen in Figure 2A, there was no difference among groups at 24 hours (p>0.05). However, at 48 hours higher proliferation of hDPSCs exposed to CaneCPI-5 than the control (p<0.05) was observed. Furthermore, there was a higher proliferation of cells exposed to CaneCPI-5 for 48h when compared to 24h (p<0.05).

### Transwell cell migration assay

At 24h, CaneCPI-5 showed a stimulatory effect on cell migration. hDPSCs treated with CaneCPI-5 showed higher migration to the lower compartment of the transwell membrane when compared to untreated cells (p<0.05) (Figures 2B and C).

### Alizarin red staining

**Figure 3:**
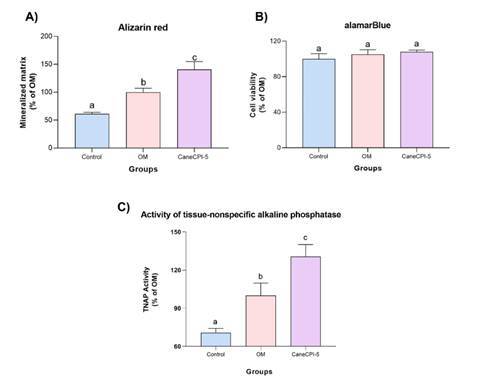
Alizarin red staining assay (A), Viability of hDPSCs (B), and Activity of tissue-nonspecific alkaline phosphatase (TNAP) (C). (A) Comparison of the production of mineralized nodules among groups. Different letters in the columns indicate significant differences between groups (one-way ANOVA and Tukey post-test. (B) The viability of hDPSCs was assessed by alamarBlue assay in control, osteogenic medium (MO), and CaneCPI-5 (0.05 μg/μL) groups. (C) Comparison of TNAP activity among groups. Different letters in columns indicate significant differences between groups (one-way ANOVA and Tukey post-test).

There was a higher formation of mineralized nodules in an osteogenic medium group compared to the control group (p<0.0001). CaneCPI-5 (0.05 μg/μL) showed a significant stimulatory effect on the formation of mineralized nodules (p<0.0001) when compared to the osteogenic medium group and the control group (Figure 3A). The alamarBlue assay, which was performed to monitor hDPSCs viability, showed that there was no difference among groups (p>0.05) (Figure 3B).

### Activity of tissue-nonspecific alkaline phosphatase (TNAP)

There was higher TNAP activity in an osteogenic medium group compared to the control group (p<0.05). CaneCPI-5 (0.05 μg/μL) induced significant TNAP activity when compared to control and osteogenic medium groups (p<0.05) (Figure 3C).

## Discussion

The null hypothesis was rejected since CaneCPI-5 promoted effects on all assessed parameters.

The human dental pulp is a source of mesenchymal stem cells ^20^, which constitute a suitable source for in vitro models to assess the potential of substances to induce mineralizing phenotype ^1^ ^,^ ^21^. One of the criteria for considering a cell as a mesenchymal stem/progenitor type is the high expression of markers such as CD105, CD73, CD90, and CD146. In parallel, there must be low or no expression of negative markers such as CD45 and CD34, considered hematopoietic and/or endothelial cell markers ^22^ ^,^ ^23^, as shown in the study by Cassiano et al. ^15^.

The low cytotoxicity of CaneCPI-5 has been previously shown in MC3t3-E1 pre-osteoblasts ^12^. CaneCPI-5, from 0.025 μg/μL to 0.2 μg/μL, did not promote cytotoxicity, which is in line with a previous study that revealed the same effect of CaneCPI-5, from 0.025 μg/μL to 1 μg/μL, in human gingival fibroblasts ^24^. Although without significant differences, cells exposed for 24 h to 0.0125μg/μL, 0.025μg/μL, and 0.05μg/μL CaneCPI-5 tended to show higher cell viability than the control group and had higher viability than cells exposed to 0.2 μg/μL. The 0.05 μg/μL concentration of CaneCPI-5 was chosen to perform the other assays since it was the highest concentration that provided high hDPSCs viability tendency by the alamarBlue assay, which was the first screening to observe the CaneCPI-5 effects on hDPSCs.

The processes of tissue regeneration and repair involve, among other factors, the migration of precursor cells, which are capable of differentiating and secreting extracellular matrix to restore the integrity and function of the injured tissue ^25^. CaneCPI-5 stimulated higher hDPSCs migration than the control group, by transwell assay. Since there is no study that used the transwell assay for hDPSCs migration in contact with CaneCPI-5, an adequate comparison with other studies is not possible.

Another fundamental aspect of tissue repair is cell proliferation ^26^. Ideally, we sought bioactive substances that promote the enhancement of this phenomenon ^27^. In this study, cell proliferation was evaluated using the BrdU colorimetric assay, which is based on the incorporation of bromodeoxyuridine during DNA synthesis in multiplying cells. The proliferation of cells treated with CaneCPI-5 was not different from the control at 24 hours; however, at 48 hours CaneCPI-5 induced higher proliferation when compared to the control group. To the best of our knowledge, this is the first study that used BrdU incorporation for hDPSCs proliferation in contact with CaneCPI-5.

Alkaline phosphatase is considered a marker of osteo/odontogenic differentiation, being one of the main enzymes expressed in the initial maturation of osteoblasts and odontoblasts, which play an important role in biomineralization. Measuring the activity of this enzyme makes it possible to evaluate material bioactivity and the potential to promote repair through the formation of mineralized tissue ^28^ ^,^ ^29^. In the present study, CaneCPI-5 induced higher activity of this enzyme in hDPSCs at 7 days compared to the positive control group (osteogenic medium). Additionally, CaneCPI-5 had the potential to induce hDPSCs differentiation into a mineralizing phenotype since there was a significantly higher formation of mineralized nodules in the CaneCPI-5 group than in the osteogenic medium (control group), at 21 days. These results are in line with a study that showed, in preosteoblasts (MC3t3-E1), that CaneCPI-5 can trigger biological mechanisms related to osteoblast differentiation. Among these mechanisms is the overexpression of genes such as Osterix and ALP, which are classical biomarkers of osteogenic phenotypes ^12^. These results are also in line with studies that have shown that the cystatin derived from sweet orange (CsinCPI-2) can induce a mineralizing phenotype ^1^ ^,^ ^13^; CaneCPI-5 and CsinCPI-2 are recombinant phytocystatins that have the same target (inhibition of cathepsins).

Considering the current observations, CaneCPI-5 constitutes a molecule with the potential to be used in endodontic regeneration techniques or pulpal and periapical repair, which are processes that require migration, proliferation, and osteo/odontogenic differentiation of mesenchymal stem cells. However, more studies evaluating the gene and protein expression of factors related to the induction of the mineralizing phenotype, among others, are necessary to understand its effect on mesenchymal stem cells, and its application. The incorporation of CaneCPI-5 in scaffolds or in a medicinal compound to be used during endodontic therapy would be interesting as it could contribute to the modulation of bone resorption through the inhibition of cathepsins and anti-inflammatory activity, as well as favoring pulpal and periapical repair/regeneration through the activation of osteo/odontogenic differentiation mechanisms.

Finally, it is important to mention that in vitro models have strengths such as maintaining well-controlled conditions, reflecting different levels of cellular organization and behavior, and even providing a certain degree of information to the clinical situation. However, the main disadvantage that limits these models is that they cannot reflect the exact in vivo physiology, which depends on crosstalk between various cell types and dynamics among them ^30^. For this reason, it is necessary that in vitro studies be complemented with in vivo studies.

## Conclusion

CaneCPI-5 was cytocompatible, and induced migration, proliferation and osteogenic differentiation in hDPSCs. Therefore, CaneCPI-5 represents a molecule with promising potential for use in endodontic therapy to stimulate events necessary for pulp and periapical repair/regeneration.
